# Highly expressed ribosomal protein L34 indicates poor prognosis in osteosarcoma and its knockdown suppresses osteosarcoma proliferation probably through translational control

**DOI:** 10.1038/srep37690

**Published:** 2016-11-24

**Authors:** Shuju Luo, Jinmin Zhao, Mitra Fowdur, Kun Wang, Tenglong Jiang, Maolin He

**Affiliations:** 1 Division of Spinal Surgery, The First Affiliated Hospital of Guangxi Medical University, No. 6 Shuangyong Road, Nanning, Guangxi, 530021, China; 2 Guangxi Key Laboratory of Regenerative Medicine, Guangxi Medical University, No. 22 Shuangyong Road, Nanning, Guangxi, 530021, China; 3 Department of Orthopedic Trauma and Hand Surgery, The First Affiliated Hospital of Guangxi, Medical University, No. 6 Shuangyong Road, Nanning, Guangxi, 530021, China

## Abstract

Osteosarcoma has devastating health implications on children and adolescents. However, due to its low incidence and high tumor heterogeneity, it is hard to achieve any further improvements in therapy and overall survival. Ribosomal protein L34 (RPL34) has been increasingly recognized to promote the proliferation of malignant cells, but its role in osteosarcoma has not been investigated. In this study, real-time quantitative PCR (RT-qPCR) and immunohistochemistry revealed that RPL34 was highly expressed in osteosarcoma tissues when compared to adjacent tissues and normal bone tissues. Survival analysis showed that high expression of RPL34 predicted a poor prognosis for osteosarcoma patients. Knockdown of RPL34 in Saos-2 cells via lentivirus-mediated small interfering RNA (siRNA) significantly inhibited cell proliferation, induced cell apoptosis and G2/M phase arrest. Moreover, screening of transcription factors using University of California Santa Cruz (UCSC) Genome Browser, protein-protein interaction (PPI) network analysis, Gene Ontology (GO) and pathway enrichment analysis revealed that MYC participates in the transcriptional regulation of RPL34, which interacts with the subunits of eukaryotic translation initiation factor 3 (eIF3) and probably involves the translational control of growth-promoting proteins. Our findings suggest that RPL34 plays an important role in the proliferation of osteosarcoma cells.

Osteosarcoma (OS) is the most prevalent primary sarcoma of bone and accounts for 35% of all primary malignant bone tumors^1^. The incidence rate of OS for all races and both sexes is 4–5 cases per year per million persons^2^. OS ranks among the most common causes of cancer-related death in the pediatric age group^3^. With the application of neoadjuvant chemotherapy and the advances of surgical techniques, the overall 5-year survival rate for OS patients has been enhanced from less than 20% to 65–70%^4^. However, the trend of survival rate have stagnated over the past 20 years^5^. Therefore, the survival plateau of OS forces us to explore new therapeutic targets and agents.

Mammalian ribosomes consist of a 40S subunit and a 60S subunit, which require the coordinated assembly of 4 different ribosomal RNAs (rRNAs) and about 80 ribosomal proteins. Ribosomal proteins are also known as ribosomal proteins in small subunit (RPS) or ribosomal proteins in large subunit (RPL) in accordance with the size of subunits they stem from. In the past, it was supposed that ribosomal proteins only perform housekeeping functions, mainly implicated in stabilizing the specific structures of rRNAs in mature subunits and facilitating the accurate folding of rRNAs during ribosome assembly^6^. Precise control of ribosomal protein synthesis is vitally important for normal ribosome biogenesis. However, researches have increasingly found that ribosomal proteins, in addition to their roles in ribosome biogenesis, also exhibit some extraribosomal functions that have not yet been extensively characterized in other cellular processes such as proliferation^7^,^8^, differentiation^9^, apoptosis^10^,^11^, development regulation^12^,^13^,^14^,^15^, cell malignant transformation^16^,^17^, DNA repair^18^ and chemoresistance^19^,^20^.

RPL34 belongs to the L34E family of ribosomal proteins and is located in cytoplasm. The gene encoding RPL34 is mapped to chromosome 4q25 and spans approximate 10 Kb (human assembly: GRCh38/hg38) with 6 exons. RPL34 with a molecular weight about 13 kDa is a highly-conserved protein mainly involved in the formation of the 60S subunit. But several studies have found that RPL34 show secondary functions in other cellular processes of different species. The process that pea axillary buds accumulate RPL34 mRNA during growth-dormancy cycles was analyzed by RNA gel blotting and Flow Cytometry (FCM). It was found that accumulation of RPL34 is correlated with cell proliferation, and that RPL34 is involved in the cell cycle regulation of pea axillary buds^21^. Research on tobacco suggested that, in contrast to mature tissues, the level of RPL34 mRNA was significantly higher in rapidly proliferating tissues, which indicated that RPL34 is related to cell proliferation in tobacco^22^. The growth of a bacillus subtilis mutant harbored a deletion of RPL34 was restrained, resulting from RPL34 deficiency^23^. Additionally, RPL34 was proven to be implicated in the regulation of polyamine biosynthesis in *Escherichia coli*^24^, which has an impact on chromsome stability, genetic transcription and protein synthesis. More notably, the dysregulated expression of RPL34 has been seen in several cancers. Liu *et al*.^25^ showed that RPL34 was highly expressed in gastric cancer cell lines, and that knockdown of RPL34 in SGC-7901 cells significantly suppressed cell proliferation, induced apoptosis and arrested cells in S phase. Yang *et al*.^26^ found that RPL34 expression was up-regulated in non-small cell lung cancer (NSCLC) tissues compared with adjacent tissues. RPL34 knockdown in NSCLC H1299 cells strongly inhibited cell proliferation, increased apoptosis and resulted in cell cycle arrest in S phase. These observations support the idea that RPL34 is closely correlated with malignant cells proliferation.

The important role of dysregulated ribosomal proteins synthesis in the proliferation of malignant cells has been validated for RPL34. Nevertheless, until now, the mechanism through which RPL34 affects the proliferation of malignant cells and the role of RPL34 in human OS cells remain largely unclear. In this study, we detected the expression level of RPL34 in human OS tissues and adjacent tissues, as well as in normal bone tissues and human OS cell lines. Then we evaluated its prognostic value in OS patients. Finally, we explored the effect of RPL34 knockdown on the proliferation of human OS cells and its underlying molecular mechanism based on lentivirus-mediated siRNA and bioinformatics methods.

## Results

### RPL34 is Frequently UP-regulated in Osteosarcoma

**Figure 1:**
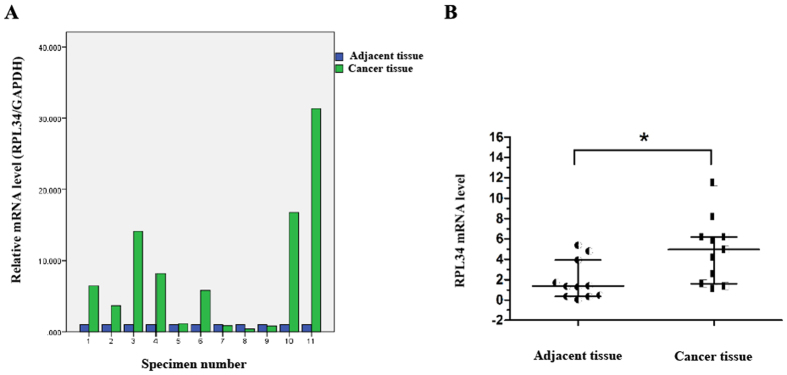
RPL34 mRNA levels in 11 pairs of human OS tissues and adjacent tissues. (**A**) The interleaved bars show the fold changes of RPL34 mRNA levels in the OS tissues, as compared to those in the adjacent tissues. (**B**) The scatter plot displays a significant up-regulation of RPL34 mRNA in the OS tissues compared with the adjacent tissues, where the longer lines represent the median with interquartile range of 2^−ΔΔCt^, *P = 0.015.

**Figure 2:**
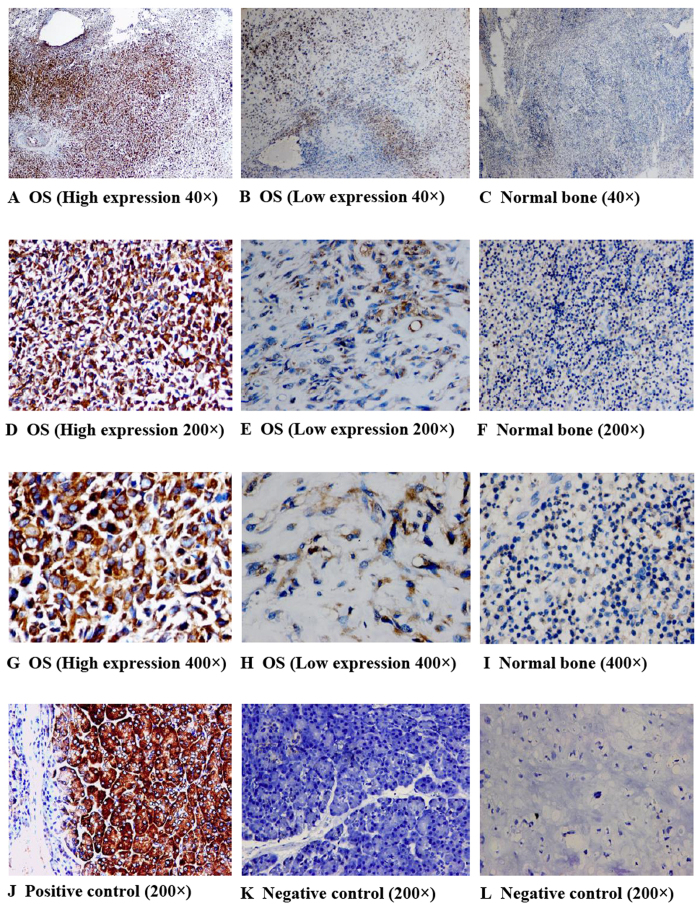
Imunohistochemical staining of RPL34 on 95 OS tissues and 60 normal bone tissues. The representative stainings of RPL34 on OS tissues and normal bone tissues, under 40×, 200× and 400× high-power fields, are showed in the figure. Osteosarcoma is abbreviated as OS. RPL34 was over-expressed in the OS tissues, as compared to the normal bone tissues, ***P = 0.000. Pancreatic tissue was used as a control for RPL34 antibody.

To investigate the effect of RPL34 expression on OS, we first determined the transcription level of RPL34 in human OS tissues and adjacent tissues by RT-qPCR, where RPL34 mRNA was shown to be up-regulated in 7/11 (63.64%) of the OS tissues at more than 3-fold higher levels (Fig. 1A). Compared with the adjacent tissues, the mRNA level of RPL34 was significantly increased in the OS tissues (Fig. 1B, P = 0.015). To confirm this result, RPL34 expression was further evaluated in 95 OS tissues and 60 normal bone tissues by immunohistochemistry analysis. Our data showed that the expression of RPL34 protein was detected in cytoplasm. According to the category standard, 75/95 (78.95%) of the OS tissues showed strongly positive staining with immunoreactive scores ranged from more than 4 to 12 and the other 20/95 (21.05%) OS tissues showed weakly positive staining with immunoreactive scores ranged from more than 0 to 4, whereas all of the normal bone tissues were weakly stained. The results revealed that RPL34 expression was also significantly up-regulated in the OS tissues, compared to the normal bone tissues (Fig. 2, P = 0.000).

### Osteosarcoma Patients with High RPL34 Expression Have Worse Clinical Outcomes

**Figure 3:**
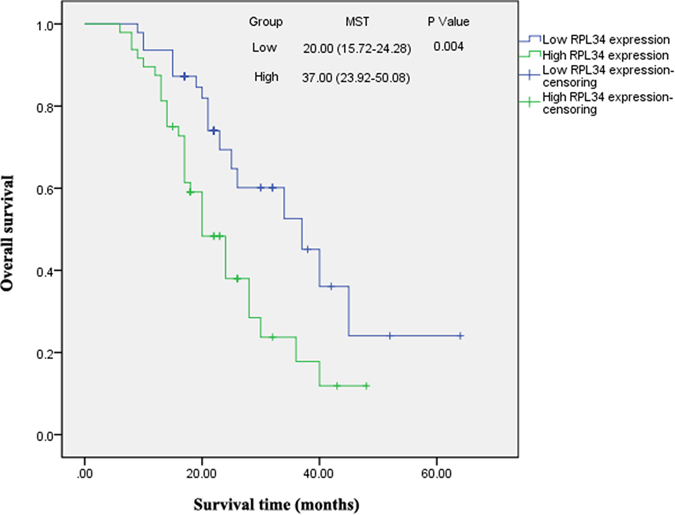
Survival analysis of 95 OS patients. Kaplan-Meter survival curves represent the overall survival for OS patients in the two groups, indicating that the overall 3-year survival rate of the OS patients with high RPL34 expression is significantly lower than those with low RPL34 expression, **P = 0.004.

All 95 patients with OS were regularly followed-up in the long term. Kaplan-Meier survival curve was created to describe the overall survival for the patients with OS. A significant difference of the overall 3-year survival rate was observed between the high-expression RPL34 patients with immunoreactive scores >7.1 (35.42%, 17/48) and the low-expression RPL34 patients with immunoreactive scores ≤7.1 (61.70%, 29/47) by log-rank test (Fig. 3, P = 0.004). The data indicated that high-level expression of RPL34 in patients with OS predicts a worse prognosis.

### Expression Levels of RPL34 in hFOB1.19 Cells and Human Osteosarcoma Cell Lines and Lentivirus-mediated siRNA Specifically inhibits RPL34 Expression

**Figure 4:**
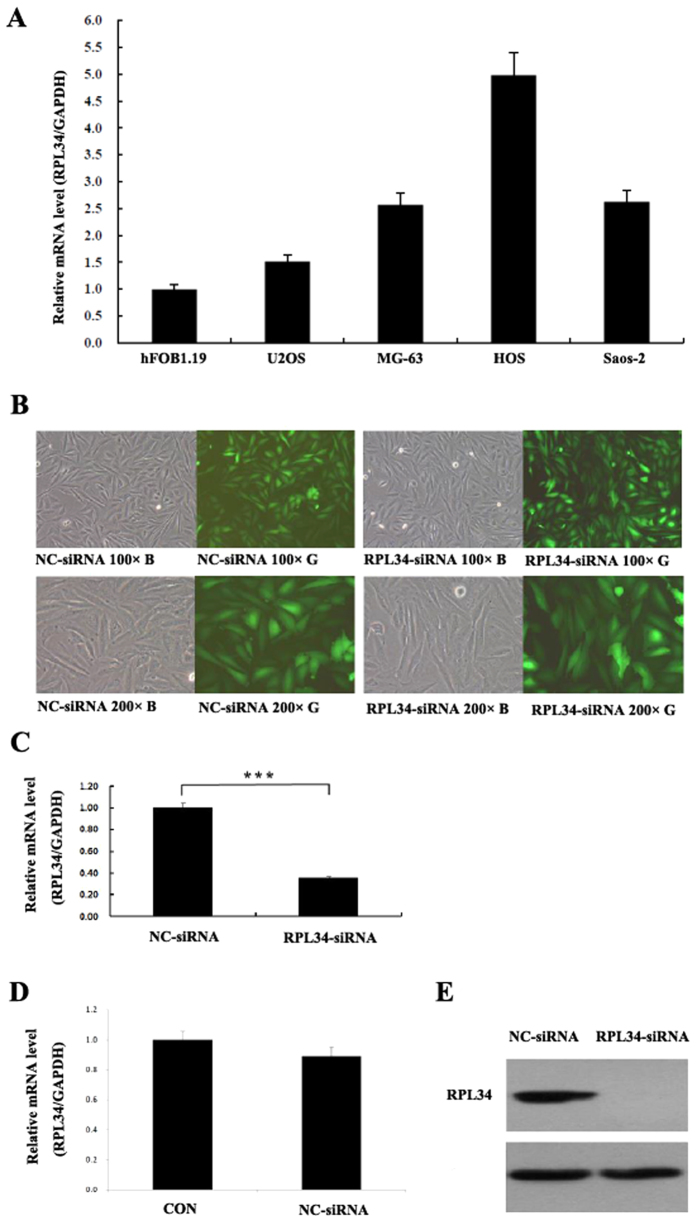
RPL34 mRNA levels in hFOB1.19 osteoblast cell line and human OS cell lines and the knockdown efficacy of RPL34 mediated by RPL34-siRNA both in mRNA and protein levels. (**A**) The mRNA levels of RPL34 in hFOB1.19 cells and U2OS, MG-63, HOS and Saos-2 cell lines were detected by RT-qPCR. GAPDH was used as an internal control. (**B**) The fluorescence microscope was used to determine the transfection efficacy of RPL34-siRNA and NC-siRNA lentivirus. Original magnification: 100× and 200×. (**C**) The mRNA level of RPL34 was significantly down-regulated in RPL34-siRNA treated group compared with NC-siRNA treated group, ***P = 0.0008. (**D**) The lentivirus vector had no effect on the expression of RPL34 in Saos-2 cells, P = 0.091. (**E**) Western blot further confirmed RPL34 protein down-regulation of Saos-2 cells in RPL34-siRNA treated group, as compared to NC-siRNA treated group.

RT-qPCR was used to determine the mRNA level of RPL34 in human osteoblast cell line hFOB1.19 and human OS cell lines U2OS, MG-63, HOS and Saos-2. A higher RPL34 mRNA level was detected in human OS cell lines compared with human osteoblast hFOB1.19 cells (Fig. 4A). To explore the effects of RPL34 knockdown on the biological behaviors of OS cells, we introduced lentivirus-mediated siRNA targeting RPL34 into Saos-2 cells. A fluorescence microscope was used to determine the transfection efficacy of the recombinant lentivirus. The observation showed that most of Saos-2 cells (>80%) were detected with GFP expression 3 days after transfection (Fig. 4B). The knockdown efficacy of RPL34 was confirmed by RT-qPCR and western blot. As shown in Fig. 4C (P = 0.0008), RPL34 mRNA level in Saos-2 cells treated with RPL34-siRNA lentivirus was decreased by about 64% as compared to those treated with NC-siRNA lentivirus 5 days after transfection, while Saos-2 cells without lentivirus transfection served as a blank control (Fig. 4D, P = 0.091). The level of RPL34 protein was also significantly reduced by RPL34-siRNA lentivirus transfection (Fig. 4E).

### Knockdown of RPL34 Significantly Suppresses Saos-2 Cells Proliferation

**Figure 5:**
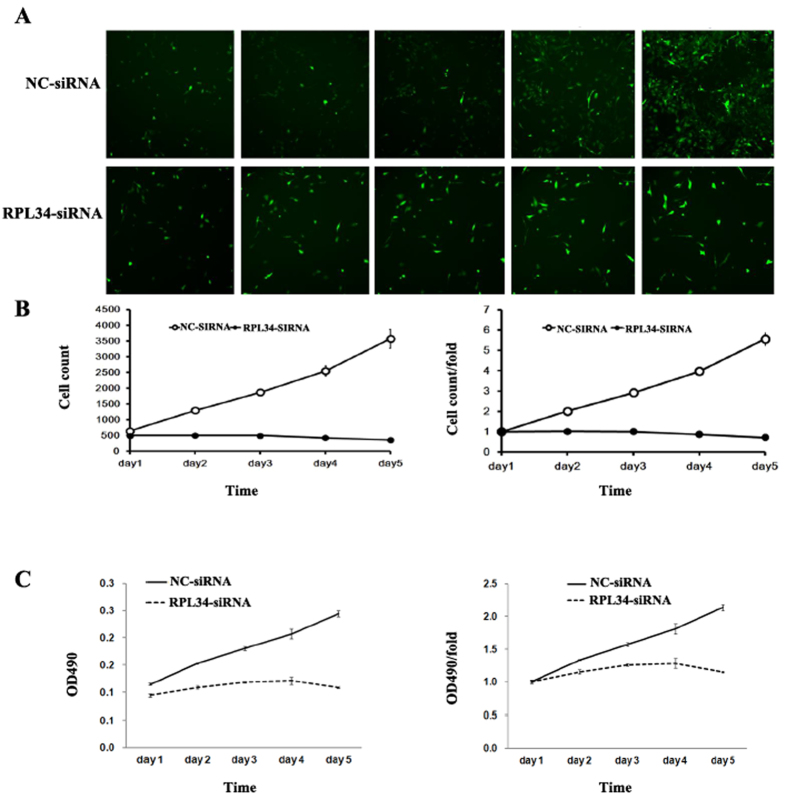
Effect of RPL34 knockdown on Saos-2 cells proliferation. (**A**) The HCS was used to observe Saos-2 cells growth for 5 consecutive days after transfection with either RPL34-siRNA lentivirus or NC-siRNA lentivirus. The growth of cells transfected with RPL34-siRNA lentivirus was significantly inhibited compared with those transfected with NC-siRNA lentivirus. (**B**) The growth curves of Saos-2 cells were compared between RPL34-siRNA treated group and NC-siRNA treated group. (**C**) MTT assay was performed to detect the proliferative capacity of Saos-2 cells separately transfected with RPL34-siRNA lentivirus and NC-siRNA lentivirus. RPL34 knockdown significantly suppressed the proliferation of Saos-2 cells.

HCS was performed to assess the effects of RPL34 knockdown on OS cells growth *in vitro*. Saos-2 cells transfected with different lentivirus were cultured in 96-well plates and the plates were read on ArrayScan^®^ VTI HCS Reader every day, which enabled automated cell images acquisition, analysis and quantification (Fig. 5A). The cell growth rate was defined as the ratio of cell count on a certain day to the cell count on the first day. The growth curves are shown in Fig. 5B. The HCS result showed that the growth rate of Saos-2 cells transfected with RPL34-siRNA lentivirus was slower than that of Saos-2 cells transfected with NC-siRNA lentivirus. As shown in Fig. 5C, a similar result was also observed in MTT assay, which further corroborated the negative effect of RPL34 knockdown on Saos-2 cells proliferation. Taken together, the results indicated that RPL34 plays a critical role in the proliferation of OS cells.

### Knockdown of RPL34 in Saos-2 Cells Leads to Inhibition of Colony Formation

**Figure 6:**
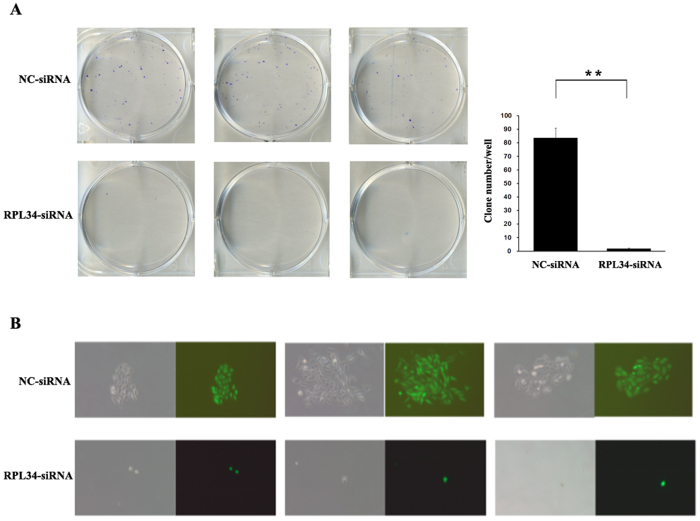
Effect of RPL34 knockdown on the colony formation of Saos-2 cells. (**A**) The colony formation of Saos-2 cells treated with either RPL34-siRNA lentivirus or NC-siRNA lentivirus was detected by the colony formation assay and data were expressed as mean ± SD. The number of colony-forming units in RPL34-siRNA treated group was remarkably less than that in NC-siRNA treated group after 10 days of incubation, **P = 0.0026. (**B**) The representative colonies of Saos-2 cells in the two groups.

To assess the effects of RPL34 knockdown on the tumorigenic ability of Saos-2 cells, we detected the colony formation capacity of Saos-2 cells transfected with either RPL34-siRNA lentivirus or NC-siRNA lentivirus. As shown in Fig. 6A, after 14 days of incubation the number of colonies in RPL34-siRNA treated group (2 ± 1) was remarkablely less than that in NC-siRNA treated group (84 ± 7) (P = 0.0026). Furthermore, the cell number of a single colony in RPL34-siRNA treated group was also less than that in NC-siRNA treated group (Fig. 6B). The results indicated that knockdown of RPL34 could significantly suppress the colony formation capacity of Saos-2 cells *in vitro*.

### Knockdown of RPL34 in Saos-2 Cells Induces G2/M Phase Arrest and Cell Apoptosis

**Figure 7:**
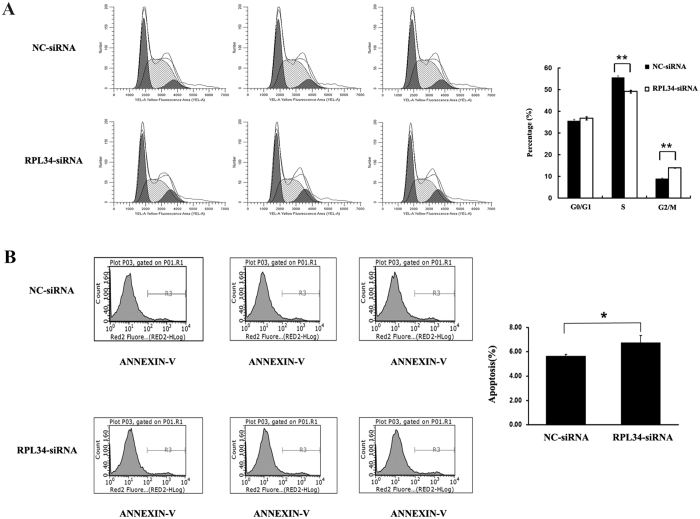
Effect of RPL34 knockdown on the cell cycle progress and apoptosis of Saos-2 cells. (**A**) The cell cycle distributions of Saos-2 cells separately transfected with RPL34-siRNA and NC-siRNA lentivirus were detected by FCM. Quantitative analysis of Saos-2 cells in different phases was performed and data were expressed as mean ± SD. RPL34 knockdown in Saos-2 cells induced G2/M phase arrest, **P(S phase) = 0.0014, **P(G2/M phase) = 0.0022. (**B**) The percentage of apoptotic cells was quantified by FCM after staining with Annexin V-APC and data were showed as mean ± SD. RPL34 knockdown in Saos-2 cells significantly increased cell apoptosis, *P = 0.042.

To confirm whether RPL34 knockdown has an impact on the cell cycle progression of OS cells, we detected the cell cycle distribution of Saos-2 cells transfected with either RPL34-siRNA lentivirus or NC-siRNA lentivirus by FCM. The cell percentages of different phases in NC-siRNA treated group were as follows: G0/G1 phase, 35.63 ± 0.76%; S phase, 55.56 ± 0.99%; G2/M phase, 8.82 ± 0.47%. Whereas those in RPL34-siRNA treated group displayed as follows: G0/G1 phase, 36.81 ± 0.74%; S phase, 49.20 ± 0.66%; G2/M phase, 13.99 ± 0.08%. RPL34-siRNA lentivirus cultures exhibited a significant decrease in the cell percentage of S phase (P = 0.0014) and an increase in that of G2/M phase (P = 0.0022) compared with NC-siRNA lentivirus cultures (Fig. 7A). The above data revealed that RPL34 knockdown could arrest Saos-2 cells in G2/M phase. To determine whether RPL34 knockdown triggers the apoptosis of OS cells, Annexin V-APC staining and FCM was used to measure the apoptotic percentage of Saos-2 cells transfected with either RPL34-siRNA lentivirus or NC-siRNA lentivirus. Notably, a significant increase in the percentage of apoptotic cells was observed in RPL34-siRNA treated group (6.73 ± 0.63%) compared with NC-siRNA treated group (5.64 ± 0.14%) (Fig. 7B, P = 0.042), which suggested that RPL34 knockdown could promote Saos-2 cells apoptosis *in vitro*.

### Transcription Factors Involved in the Regulation of RPL34 and Enrichment Analysis of the Genes Identified by PPI Network

**Figure 8:**
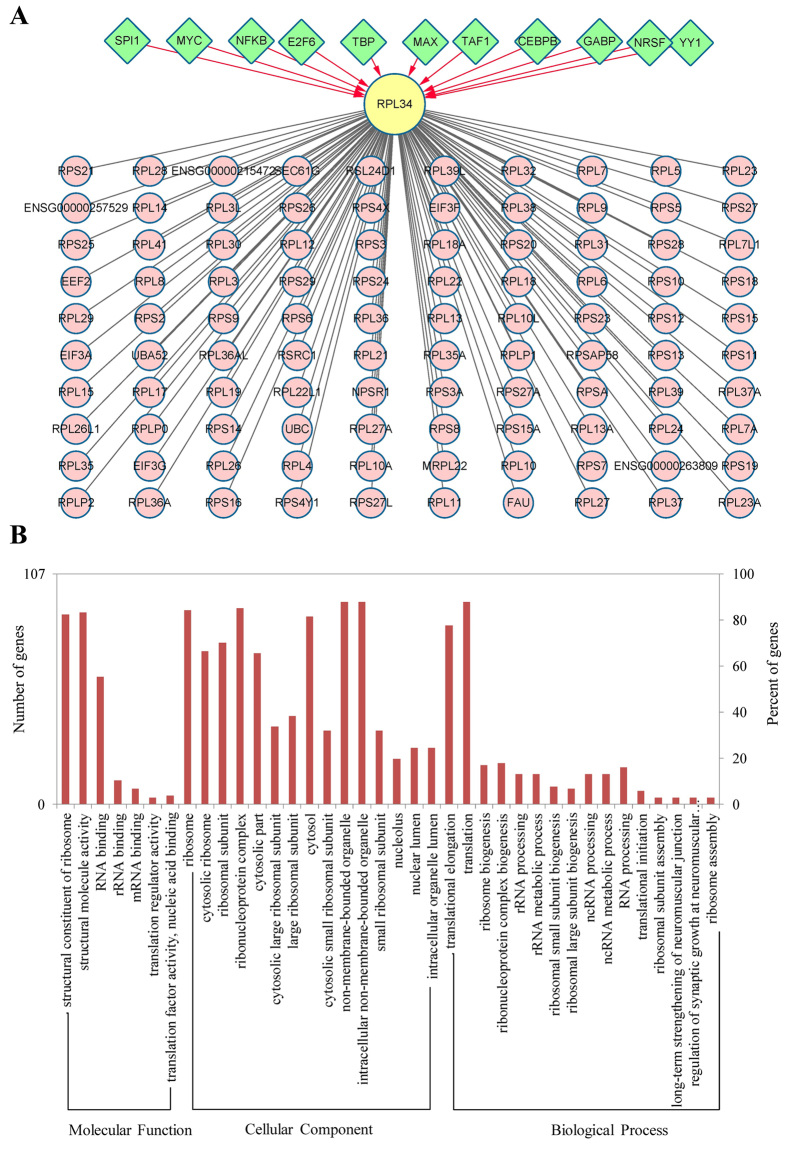
Integrated network and enrichment analysis. (**A**) Integrated network of TF-target and PPI. The TFs implicated in the transcriptional regulation of RPL34 and the proteins interacted with RPL34 are presented in green nodes and pink nodes, respectively. (**B**) GO analysis of the genes identified in PPI network. The enrichment of the genes associated with RPL34 in molecular function, cellular component and biological process revealed that RPL34 mainly involves in translational control, apart from ribosome biogenesis, probably through the interactions with the subunits of eIF3.

The transcription factors (TFs) implicated in the transcriptional regulation of RPL34 were predicted based on UCSC database. The results indicated that eleven identified TFs, particularly MYC and MYC associated factor X (MAX), were involved in the transcriptional regulation of RPL34 (Fig. 8A). The MYC oncoprotein and MAX are frequently involved in cell proliferation, differentiation and apoptosis in the form of dimers. The PPI network constructed based on the information from STRING database composes of 112 nodes and 4669 interactions (Fig. 8A and Supplementary Table S1). GO and Kyoto Encyclopedia of Genes and Genomes (KEGG) pathway enrichment analysis showed that the genes in PPI network were significantly enriched in a pathway known as ribosome and GO terms of different categories, namely molecular function (MF), cellular component (CC) and biological process (BP). The top 7 (or top 15) enriched GO terms of each category were listed in Table 1. The data indicated that the genes associated with RPL34 were mainly enriched in the GO terms related to structural constituent of ribosome and synthetic process of protein (Fig. 8B). Interestingly, each GO category, as MF termed translation factor activity and nucleic acid binding, CC termed eukaryotic translation initiation factor 3 complex and BP termed translational initiation, was significantly enriched by three identified genes in the PPI network, including EIF3A, EIF3G and EIF3F. The proteins encoded by these three genes are different subunits of the 13-subunit complex, namely eIF3. More importantly, specific individual subunits of eIF3 have been shown to be dysregulated in a wide range of human tumors.

## Discussion

Ribosome biogenesis consists of rRNA gene transcription, modification of rRNA and ribosome assembly. The regulation of the three steps is a highly coordinated process that enable precise initiation and regulation of protein synthesis, an anomaly of which is increasingly recognized to play a crucial role in cell malignant transformation and oncogenesis^27^,^28^,^29^. Besides, any dysregulation of the steps can cause an anomalous pattern of protein synthesis, especially a specific programme of proteins associated with cell growth control, thus leading to an increase of susceptibility to tumorigenesis^11^. In particular, the ribosome assembly involves the association of rRNAs and ribosomal proteins, and ectopic production or activation of ribosomal proteins have been actually observed in several cancer types^30^,^31^, such as RPS6 in NSCLC and non-Hodgkin lymphoma^32^,^33^; RPS7, RPS15A and RPS20 in colorectal cancer^34^,^35^,^36^; RPL5 and RPL10 in T-cell acute lymphoblastic leukemia^37^; and RPS14 in 5q- syndrome^38^, a subtype of myelodysplastic syndrome (MDS) with a high propensity to acute myeloid leukemia (AML).

Several ribosomal proteins also have been shown to have a role in oncogenesis and progression of OS. Knockdown of RPS15A by lentivirus-mediated RNAi system significantly suppressed the proliferation of human OS U2OS cells and led to G0/G1 phase arrest^39^. The expression levels of RPS3 were increased in human OS cell lines and OS tissues compared with those in osteoblast cells and normal bone tissues. Furthermore, RPS3 expression was also up-regulated in primary OS tissues of patients with lung metastasis compared with those without. Notably, RPS3 was identified as a novel target of GLI2, which involves in the invasion and metastasis of OS^40^. In addition, it was reported that RPL7A expression was significantly down-regulated in OS tissues compared with that in normal bone tissues and benign bone lesions both in mRNA and protein levels. Low expression of RPL7A indicated a poor clinical outcome for overall survival in OS patients with lung metastasis at the time of initial diagnosis with primary OS^41^.

RPL34 is an evolutionarily highly conserved protein. However, in recent years, an increasing number of researches have associated RPL34 with the extraribosomal functions related to cellular proliferation^31^. More importantly, several reports have showed that RPL34 plays a critical role in the proliferation, cell cycle progression and apoptosis of malignant cells^25^,^26^. Nonetheless, its role in OS has been poorly characterized. Here, we identified RPL34, which has been confirmed to be highly associated with the proliferation and prognosis of OS, as an underlying attractive novel therapeutic target for OS. To our knowledge, the results of the present study have shown for the first time that RPL34 mRNA expression was significantly up-regulated in OS tissues compared with that in adjacent tissues. A similar result was observed in the immunohistochemical stainings of OS tissues and normal bone tissues. Moreover, the current research analyzed the prognostic value of RPL34 expression in OS and first revealed that high-expression RPL34 patients suffered from a significantly poorer overall survival than low-expression RPL34 patients did. In addition, our data showed that knockdown of RPL34 by lentivirus-mediated siRNA in human OS Saos-2 cells resulted in a significant inhibition of proliferation, an increase of apoptosis and G2/M phase arrest, part of which is in conformity with the research results in human gastric cancer and NSCLC. Our findings further strengthen the evidence that supports the positive effect of high RPL34 expression on the proliferation of malignant cells and its pro-tumorigenic role.

Unfortunately, the detailed molecular mechanisms through which RPL34 mediates the increased proliferative ability of malignant cells remain largely unknown up to now. In the present study, we obtained eleven putative TFs that may involve in transcriptional regulation of RPL34 based on the integrated analysis of human TF-gene pairs from UCSC database. Actually, several oncoproteins or tumor suppressors have been proven to be implicated in the control of protein synthesis through the transcriptional regulation of ribosomal proteins so as to affect tumorigenesis or progression of cancer^6^,^31^,^42^, and of particular note among those TFs identified in our study is MYC, a well characterised oncogene^43^, enhanced expression or amplification of which has been reported in numerous cancer types, including OS^44^. As we all know, MYC functions as a TF via interacting with MYC associated factor X that was also found to be implicated in transcriptional regulation of RPL34 in our study, and its amplification has been correlated with oncogenesis, proliferation and metastasis of OS^44^,^45^. Additionly, we also found that three subunits of eIF3 (3a, 3g, 3f) might interact with RPL34 based on GO and pathway enrichment analysis of the identified genes in the established PPI network. eIF3, as the largest and most complex translational initiation factor, comprises of 13 non-identical subunits named as eIF3a-eIF3m, and play a key role in multiple steps during the initiation phase of protein synthesis. Although the detailed mechanisms to explain the involvement of eIF3 subunits in oncogenesis have not been well illuminated, strong evidence suggested that the individual overexpression of 6 non-identical eIF3 subunits as 3a, 3b, 3c, 3h, 3i and 3m lead to malignant transformation, and low-expression of 2 other subunits as 3e and 3f cause a similar result^46^. EIF3a is the largest subunit and one of the core subunits of eIF3, and its elevated expression compared to adjacent tissues is seen in a wide range of human cancers^46^. High level of eIF3a promotes cell proliferation and malignancy probably through the translational control of specific mRNAs, such as ribonucleotide reductase regulatory subunit M2 (RRM2), N-myc downstream regulated 1 (NDRG1) and cyclin-dependent kinase inhibitor 1B (CDKN1B)^47^,^48^,^49^. In contrast, reduced expression of eIF3f compared with adjacent tissues have been found in melanoma and pancreatic cancer tissues^50^,^51^,^52^. And the eIF3f subunit was showed to be phosphorylated by the cyclin-dependent kinase 11 (CDK11p46), which increases its binding to the eIF3 complex, consequently affecting the translation initiation control, apoptotic signaling and cell malignant transformation^53^,^54^. Moreover, a recent study further revealed that eIF3 can function as either a translational activator or repressor of cap-dependent mRNAs through binding to these mRNAs via a conserved secondary structure known as RNA stem loop^55^. However, whether RPL34 is transcriptionally regulated by MYC and interacts with the subunits of eIF3 subsequently involving in the translational control of a specific repertoire of mRNAs encoding growth-promoting proteins, remains to be proved through further experiments.

## Methods

### Tissue Specimens and Cell Lines

Eleven pairs of human OS tissues and adjacent tissues for RT-qPCR were obtained from the patients in surgery who were pathologically diagnosed as OS at the First Affiliated Hospital of Guangxi Medical University. A total of 155 formalin-fixed and paraffin-embedded specimens from the pathology department including 95 OS tissues and 60 normal bone tissues were used for immunohistochemical staining. All 95 patients had received no radiotherapy and chemotherapy prior to surgery, and underwent serial monitoring every 3 months within the first 2 years after surgery and semiannually thereafter. The deadline for follow-up was October 1, 2015 and the survival status was confirmed by periodic out-patient re-examination and telephone follow-up. The use of tissue specimens in this study were approved by the research ethics committee of the First Affiliated Hospital of Guangxi Medical University, and were carried out in accordance with the relevant guidelines and regulations of the First Affiliated Hospital of Guangxi Medical University. Additionally, informed written consents were obtained from all participants involved in this study. Human osteoblast cell line hFOB1.19, human OS cell lines U2OS, MG-63, HOS, Saos-2 and human renal epithelial 293T cells were purchased from the Cell Bank of Type Culture Collection of Chinese Academy of Sciences (CBTCCCAS, Shanghai, China) and cultured in RPMI-1640 medium (Gibco, Grand Island, US) and DMEM medium (Invitrogen, Carlsbad, US) supplemented with 10% fetal bovine serum (FBS).

### Real-Time Quantitative PCR

Total RNA was extracted using the TRIzol reagent (Invitrogen, Carlsbad, US). 2 ug of total RNA from each specimen was reverse-transcribed into cDNA with M-MLV RT Kit (Promega, Madison, US). 1 ul cDNA was used as a template for PCR, which was performed by two-step method using SYBR Master Mix (Takara, Osaka, Japan) with 60 °C annealing temperature and 45 amplification cycles. GAPDH was used as an internal control. The primers used for PCR were as follow: RPL34-F GTTTGACATACCGACGTAGGC, RPL34-R GCACACATGGAACCACCATAG; GAPDH-F TGACTTCAACAGCGACACCC, GAPDH-R CACCCTGTTGCTGTAGCCAAA. The lengths of amplified fragments for RPL34 and GAPDH were 241bp and 121bp, respectively.

### Immunohistochemical Staining and Survival Analysis

The dewaxing was achieved by soaking paraffin-embedded tissue sections in xylene gradient solvent for 20 minutes and then in gradient ethyl alcohol for another 20 minutes. Heat-mediated antigen retrieval was performed on each section with citrate buffer (pH6.0) for 3 minutes. 3% H_2_O_2_ was added on top of the sections, proceeding for 10 minutes, to quench endogenous peroxidase activity. The sections were incubated with 10% goat serum (Zhongshan Biotechnology, Beijing, China) for 15 minutes to block non-specific binding sites. Rabbit anti-RPL34 antibody (1:20, Abcam, Cambridge, UK, Cat.No.GR99144-7) was added to incubate the tissues overnight at 4 °C. The horseradish peroxidase-conjugated secondary antibody (Zhongshan Biotechnology, Beijing, China, Cat.No.WP142714) was added on top of the sections incubating for 15 minutes at room temperature. DAB (Zhongshan Biotechnology, Beijing, China) was used as chromogenic agent, and hematoxylin was added to counterstain the nuclei of cells for 1 minute. The sections was placed in a solution containing 1% HCl and 99% ethanol for 10 s and then was transferred into water for 8 minutes, followed by air-dry for 20 minutes.

The staining results were observed under a microscope. We randomly selected 10 high-power fields (×400) to score the staining intensity and the positive cell percentage. The immunoreactive score (values, 0–12) was determined by the product of the score for staining intensity and the score for positive cell percentage. The staining intensity was scored as follows: 0 (non-staining); 1 (pale brown); 2 (brown); 3 (dark brown). The positive cell percentage was scored as follows: 1 (≤10%); 2 (range from more than 10% to 50%); 3 (range from more than 50% to 80%); 4 (>80%). For statistical analysis, the staining results were defined in accordance with the immunoreactive scores as follows: negative (0); weakly positive (range from more than 0 to 4); strongly positive (range from more than 4 to 12). Additionally, as the average immunoreactive score of the 95 OS specimens is 7.1, the specimens with scores ≤7.1 were categoried into low expression group and those with scores >7.1 were categoried into high expression group. Survival analysis was performed using Kaplan-Meier method and log-rank test.

### Construction of Recombinant Lentiviral Vector and Cell Infection

The siRNA (CACAGAGTCAGAAAGCTAA) targeting the RPL34 transcript (GenBank accession No. NM_000995.4) was designed and the scamble sequence (TTCTCCGAACGTGTC ACGT) was used as a lenti-negative control (NC). The short hairpin RNA (shRNA) targeting RPL34 was designed and synthesized by GeneChem Co.Ltd (Shanghai, China) based on the siRNA sequence, and the shRNA sequence was inserted into linearized vector GV115 carrying green fluorescent protein (GFP) gene and AgeI/EcoRI sites (GeneChem Co.Ltd, Shanghai, China) to construct the Lenti-shRNA vector, which were then co-transfected into 293T cells with lentiviral helper plasmids pHelper 1.0 and pHelper 2.0 (GeneChem Co.Ltd, Shanghai, China) using Lipofectamine 2000 (Invitrogen, Carlsbad, US). Lentivirus particles were obtained as previously described^56^. The RPL34-siRNA and NC-siRNA lentivirus were separately used to transfect Saos-2 cells based on the multiplicity of infection (MOI). The transfection efficacy of the recombinant lentivirus was detected by micropublisher 3.3 RTV fluorescence microscope (Olympus, Tokyo, Japan) 3 days after transfection, while the knockdown efficacy of RPL34 was determined by RT-qPCR and western blot 5 days after transfection.

### Western Blot Analysis

The expression vectors of RPL34-Flag tag fusion gene were purchased from GeneChem Co.Ltd (Shanghai, China) and then were co-transfected into 293T cells with RPL34-siRNA and NC-siRNA expression vectors, respectively. The cells were harvested and lysed with pre-cooling lysis buffer (100 mM Tris-HCl [pH 6.8], 2% β mercaptoethanol [BME], 20% Glycerin and 4% Sodium Dodecyl Sulfate [SDS]) for 10 minutes on ice. The supernatants were collected after the lysates were centrifuged at 12000 rpm at 4 °C for 15 minutes. BCA Protein Assay Kit (HyClone-Pierce, Rockford, US) was used to detect the protein concentration. The same amount of protein (20 ug) from each specimen was separated with 10% SDS-PAGE based on Laemmli buffer system^57^ and then transferred onto polyvinylidene difluoride (PVDF) membranes, which were soaked in skim milk for 60 minutes at room temperature. The PVDF membranes were incubated with mouse anti-FLAG antibody (1:3000, Sigma-Aldrich^®^ Co.LLC., Saint Louis, US) overnight at 4 °C and then with goat anti-mouse IgG antibody (1:5000, Santa Cruz Biotechnology, Santa Cruz, US) for 120 minutes at room temperature. The enhanced chemiluminescence assay was performed with ECL-PLUS/Kit (Amersham, Piscataway, US). All values were normalized against GAPDH values.

### Cell Proliferation Assay

Cell growth was detected with cellomics high content screening (HCS). After transfection with either RPL34-siRNA lentivirus or NC-siRNA lentivirus, Saos-2 cells in exponential phase were seeded in 96-well plates with 1 × 10^3^ cells per well and then were cultured at 37 °C with 5% CO2 for 5 days. The Cellomics^®^ArrayScan^®^ VTI HCS reader (Thermo Scientific™, Waltham, US) was used to scan the plates at the same time each day. The amount of cells expressing GFP was quantified with HCS Studio™ 2.0 Cell Analysis Software (Thermo Scientific™, Waltham, US). The cell growth curve was drawn for each group. For MTT assay, Saos-2 cells were collected 10 days after transfection with either RPL34-siRNA lentivirus or NC-siRNA lentivirus and then were cultured in 96-well plates at 2 × 10^3^ cells per well, followed by incubation at 37 °C with 5% CO2 for 5 days. 10 ul MTT (5 mg/ml, Beijing Dingguo Changsheng Biotechnology Co.Ltd, Beijing, China) was added to each well. The cells were incubated in the dark for 4 hours and then the medium was removed before adding 100 ul DMSO (Sinopharm Chemical Reagent Co.Ltd, Shanghai, China) to each well. The optical density was measured at 490 nm by Biotek Elx800 ELIASA (BioTek, Winooski, US).

### Flow Cytometry Analysis

FCM analysis was performed to detect cell cycle distribution and apoptosis. Saos-2 cells transfected with either RPL34-siRNA lentivirus or NC-siRNA lentivirus were incubated in 6-well plate. When the cells reached 85% confluence, the medium was removed, after which the cells were suspended, centrifuged and fixed with precooled 70% ethanol for an hour. The supernatant was removed after further centrifugation. The cells were washed with ice-cold PBS and then treated with the staining solution (40× propidium iodide [PI; 2 mg/ml, Sigma-Aldrich^®^ Co.LLC., St. Louis, US]: 100 × RNase A [10 mg/ml, Fermentas^®^, Vilnius, Lithuania]: 1 × PBS = 25: 10: 1000). The cell suspension was filtered through a 300-mesh and the cell cycle was analyzed by FACSCalibur™ Flow Cytometer (BD Biosciences, Franklin Lakes, US). As for apoptosis assay, Saos-2 cells were collected and washed with PBS 5 days after transfection. The cell suspension was centrifuged and the cells were collected after washing with binding buffer, followed by adding staining buffer to adjust the cell concentrations at 1 × 10^6^/ml. 100 ul cell suspension was stained with 5 ul Annexin V-APC (eBioscience, San Diego, US) in the dark. The apoptotic cells were detected by FCM.

### Colony Formation Assay

Colony formation assays were performed using Saos-2 cells transfected with either RPL34-siRNA lentivirus or NC-siRNA lentivirus. Saos-2 cells in exponential phase were seeded at 600 cells per well in 6-well plates. These plates were incubated for 10 days or until the colonies became clearly visible to the eyes. Next, the cells were fixed with paraformaldehyde (Sangon Biotech Co., Ltd, Shanghai, China) for 30 minutes, followed by being stained with GIEMSA (Chemicon International, Temecula, US) for 20 minutes. Then quantification of the colonies was performed using the micropublisher 3.3RTV fluorescence microscope (Olympus, Tokyo, Japan).

### Transcription Factors Screening and PPI Network Construction by Bioinformatics Technique

TFs that regulate RPL34 were screened on the basis of human TF-gene pairs obtained from the UCSC ENCODE Genome Browser (http://genome.ucsc.edu). To perform network analysis upon proteins that interact with RPL34, a PPI network was constructed via retrieving high-confidence protein interactions from STRING database^58^. The PPI combined score >0.9 was set as the threshold for interaction pairs screening. The corresponding network was visualized by Cytoscape software.

### Gene Ontology and Pathway Enrichment Analysis of the Genes Identified by PPI Network

We used DAVID to carry out GO and KEGG pathway enrichment analysis for the genes identified by the PPI network. The threshold of p value < 0.05 and the number of enriched genes ≥2 were selected as the screening criterions to choose the significantly enriched GO terms or KEGG pathways.

### Statistical Assay

Student’s t-test was used for quantitative comparison between different groups. Chi-square test and Fisher’s exact test was used for analysis of qualitative data. Mann-Whitney U test was used for comparison of immunoreactive scores. All statistical analyses were performed using the SPSS 20.0 software package (Chicago, US) and the GraphPad 5.0 Prism software package (San Diego, US). A value of P < 0.05 was accepted as statistically significant.

## Additional Information

**How to cite this article**: Luo, S. *et al*. Highly expressed ribosomal protein L34 indicates poor prognosis in osteosarcoma and its knockdown suppresses osteosarcoma proliferation probably through translational control. *Sci. Rep.* **6**, 37690; doi: 10.1038/srep37690 (2016).

**Publisher's note:** Springer Nature remains neutral with regard to jurisdictional claims in published maps and institutional affiliations.

## Supplementary Material

Supplementary Table S1

## Figures and Tables

**Table 1:** Patrt of GO terms and pathway significantly enriched by the genes associated with RPL34.

GO-ID	Category	Term	Count	*p*-value	Benjamini
GO:0003735	GOTERM_MF_FAT	structural constituent of ribosome	88	6.82E-162	6.96E-160
GO:0005198	GOTERM_MF_FAT	structural molecule activity	89	7.53E-105	3.84E-103
GO:0003723	GOTERM_MF_FAT	RNA binding	59	1.77E-47	6.00E-46
GO:0019843	GOTERM_MF_FAT	rRNA binding	11	7.44E-15	1.90E-13
GO:0003729	GOTERM_MF_FAT	mRNA binding	7	1.06E-05	2.16E-04
GO:0045182	GOTERM_MF_FAT	translation regulator activity	3	2.04E-02	2.96E-01
GO:0008135	GOTERM_MF_FAT	translation factor activity, nucleic acid binding*	4	3.89E-02	4.39 E-01
GO:0005840	GOTERM_CC_FAT	ribosome	90	1.10E-153	6.04E-152
GO:0022626	GOTERM_CC_FAT	cytosolic ribosome	71	1.73E-149	4.77E-148
GO:0033279	GOTERM_CC_FAT	ribosomal subunit	75	1.67E-136	3.06E-135
GO:0030529	GOTERM_CC_FAT	ribonucleoprotein complex	91	1.27E-116	1.74E-115
GO:0044445	GOTERM_CC_FAT	cytosolic part	70	9.54E-116	1.05E-114
GO:0022625	GOTERM_CC_FAT	cytosolic large ribosomal subunit	36	1.83E-73	1.68E-72
GO:0015934	GOTERM_CC_FAT	large ribosomal subunit	41	2.35E-70	1.84E-69
GO:0022627	GOTERM_CC_FAT	cytosolic small ribosomal subunit	34	1.45E-65	8.89E-65
GO:0015935	GOTERM_CC_FAT	small ribosomal subunit	34	5.95E-55	2.97E-54
GO:0005730	GOTERM_CC_FAT	nucleolus	21	3.65E-07	1.67E-06
GO:0031981	GOTERM_CC_FAT	nuclear lumen	26	9.88E-05	4.18E-04
GO:0070013	GOTERM_CC_FAT	intracellular organelle lumen	26	2.16E-03	8.46 E-03
GO:0031974	GOTERM_CC_FAT	membrane-enclosed lumen	26	3.85E-03	1.32 E-02
GO:0005852	GOTERM_CC_FAT	eukaryotic translation initiation factor 3 complex*	3	5.95E-03	1.91 E-02
GO:0005844	GOTERM_CC_FAT	polysome	3	8.54E-03	2.59 E-02
GO:0006414	GOTERM_BP_FAT	translational elongation	83	1.39E-175	5.26E-173
GO:0006412	GOTERM_BP_FAT	translation	94	2.20E-144	4.17E-142
GO:0042254	GOTERM_BP_FAT	ribosome biogenesis	18	2.67E-17	3.38E-15
GO:0022613	GOTERM_BP_FAT	ribonucleoprotein complex biogenesis	19	1.29E-15	1.26E-13
GO:0006364	GOTERM_BP_FAT	rRNA processing	14	1.58E-13	1.20E-11
GO:0016072	GOTERM_BP_FAT	rRNA metabolic process	14	2.79E-13	1.77E-11
GO:0042274	GOTERM_BP_FAT	ribosomal small subunit biogenesis	8	3.62E-13	1.96E-11
GO:0042273	GOTERM_BP_FAT	ribosomal large subunit biogenesis	7	3.25E-11	1.54E-09
GO:0034470	GOTERM_BP_FAT	ncRNA processing	14	1.42E-09	5.96E-08
GO:0006396	GOTERM_BP_FAT	RNA processing	17	3.10E-06	1.07E-04
GO:0006413	GOTERM_BP_FAT	translational initiation*	6	2.12E-05	6.70E-04
GO:0042257	GOTERM_BP_FAT	ribosomal subunit assembly	3	1.68E-04	4.89 E-03
GO:0042255	GOTERM_BP_FAT	ribosome assembly	3	2.44E-03	5.61 E-02
GO:0006917	GOTERM_BP_FAT	induction of apoptosis	7	3.40E-02	3.74 E-01
GO:0048638	GOTERM_BP_FAT	regulation of developmental growth	3	4.89E-02	4.10 E-01
**KEGG-ID**	**Category**	**Pathway**	**Count**	***p*****-value**	**Benjamini**
hsa03010	KEGG_PATHWAY	Ribosome	85	2.04E-171	1.63E-170

^*^Terms significantly enriched by EIF3A, EIF3G and EIF3F.
